# Editorial Notes

**Published:** 1902-09

**Authors:** 


### Editorial Notes.

The Coronation is over, the King is crowned. Long live the King! Surely it must be the first time in the history, not only of this but of all other countries, that the operation for appendicitis has postponed a Coronation, and that the Coronation would probably never have been possible but for the operation. The King and his subjects have every reason to be grateful to the surgeons who have so skilfully conducted him through the grave perils of appendicitis, and the profession generally cannot do otherwise than congratulate Sir Frederick Treves on the presence of mind and skill which enabled him to guide his Majesty through so grave a crisis. No surgeon could ever have had a more trying time than Sir Frederick during the half-hour or so preceding the finding of the pus, and what must have been his feeling of satisfaction on finding it we can only surmise: from that moment the life of the King was practically saved, and the whole nation must rejoice that no untoward accident has occurred to imperil or postpone his Majesty's recovery.

It is characteristic of so brave a patient that he should have wished to learn all there is to know of the history and pathology of his own malady, and that visit of his under the guidance of Sir Frederick Treves to the Museum of the Royal College of Surgeons shows that the conferring of the degree of F.R.C.S. has imbued his Majesty with a true scientific ardour. There is already abundant evidence that the King will continue to be one of the best of friends to the Royal Colleges of Physicians and Surgeons, whose roll is honoured by the presence of his august name. His Coronation gift to the hospitals, the King Edward's Hospital Fund, the King's Sanatorium for Consumptives, and the gift of Osborne House as a sanatorium for the officers of the navy and army, are recent illustrations of the keen interest which his Majesty takes in the work of our profession.

We are informed that the King is now in fact quite well. It is to be hoped that we shall hear no more of the inventions, which have come to us mostly from the West, that a second operation will be required to cure the appendicitis, and several other operations for the cure of cancer in the larynx and elsewhere. He has taken his turn in the *rôle* of patient, has acquitted himself with consummate fortitude, and has fairly earned immunity for many years to come.

The City of Manchester must be congratulated on having arranged one of the most successful annual meetings of the British Medical Association of recent years. The meeting was worthy of one of the great provincial centres of medical teaching and practice, and it has demonstrated very clearly that it can and does provide the high standard of scientific and practical work that is now always associated with the best British Universities. The number of distinguished foreign guests who were present, and who took an active share in the work of the sections, gave an almost international character to the discussions.

The generous hospitality of the Lord Mayor and many private citizens greatly aided the complete success of the gathering, and the President will doubtless feel that the medical profession in Manchester has been well supported in its efforts on behalf of the Association.

There was some cause for regret in the perhaps unavoidable distances between many of the various hotels and lodgings and Owens College, where the meetings were mostly held, and we venture to suggest that in future some indication of the relative localities mentioned in the advance lists of lodgings and hotels given in the British Medical Journal should be indicated by a simple sketch or map of the town or city where a meeting is about to be held.

The Royal Infirmary of Manchester is externally most gloomy and forbidding; and though efforts have been made to bring the interior up to date as far as possible, we cannot help feeling that the governors should be enabled to carry out their suggested rebuilding scheme, and provide for the most important hospital in their city a modern and more suitable building. One feature of student life there compares most favourably with that of our own Bristol students. We refer to athletics; for it was a pleasant surprise to find that they had large and well-kept grounds where cricket, tennis, hockey, football, &c., occupy the spare hours for recreation of a large number of the alumni. Perhaps if the University College, Bristol, could have a ground provided for similar purposes it would be less difficult to kindle greater enthusiasm amongst our students generally for healthy out-door sports.

The number of sectional meetings is now great, but at Manchester the attendance can only be described as having been good at very few. The medical and surgical sections were, as is usually the case, well patronised, and the discussions were perhaps less desultory than they not infrequently prove to be. An interesting discussion in the surgical section was the treatment of inoperable cancer. Apparently, although the value of the light treatment in rodent ulcer is undoubted, in other forms of carcinoma the general opinion was that it is of little benefit. One may perhaps be surprised by the view expressed by Freund and others at the section on the treatment of skin diseases that the X-rays have no bactericidal power. In the section of public health and also in the section of ethics, a somewhat novel feature was the reading of papers by gentlemen who were not members of the medical profession. The papers will no doubt be read in the pages of the *Journal*, but the method of delivery of those given before the public health section was said to have been worthy of a far larger audience than was present to hear them.

Our colleague, Dr. E. Markham Skerritt, had the honour conferred upon him of being appointed to the office of Treasurer of the British Medical Association, and the present is a somewhat critical time in the history of the Association when the presence of good judgment and a sound knowledge of finance in its treasurer are more than usually needful. We venture to think that it would be difficult to find anyone better fitted than Dr. Skerritt for the work he has accepted, and we hope that the responsibilities he undertook at Manchester, superadded to the numerous duties which he is called upon to perform in our own city and neighbourhood, will not prove to be unduly onerous.

The annual report of the Medical Officer of Health for Bristol contains much interesting matter. The large amount of work done in the laboratory is worthy of especial recognition, and we note that in 1901 a sum total of close upon three thousand examinations for the establishment of the diagnosis of diphtheria and enteric fever were made in the offices of the medical officer. This large number is mainly due to the active steps taken to follow up diphtheria contacts at school or at home; but this kind of work has become a necessary adjunct to any efficient health department, and should be performed by special assistants in a fully-equipped municipal laboratory. It is surprising to find that this work should be spoken of as "voluntary," and is not part of the recognised duties of the officers of the health department of the city. Such bacteriological work has become an absolute necessity to the general medical practitioner, who has neither the time nor the ability to carry out such researches in his consulting-room or surgery. It should not be left to private enterprise or to the voluntary goodwill of the officers of the department to provide a well-equipped laboratory, where such examinations may be made as are necessary to the diagnosis of infectious maladies.

**The Asylum, Fishponds.**—As our City and County Asylum at Fishponds is now in its last stage of development, its accommodation having reached, by reason of the recent extensions, to within 50 of the 1,000 limit, it may be of interest to review at a glance the various phases in the growth of the building.

Opened in 1861 with accommodation for 200 patients, it has been added to as follows:

| | | | | | |
|---|---|---|---|---|---|
| In 1861 | number provided for | 200, | the population of Bristol | being | 154,093 |
| 1869 | increase of | 36 | ,, | ,, | 154,093 |
| 1878 | ,, | 120 | ,, | ,, | 186,428 |
| 1888 | ,, | 437 | ,, | ,, | 206,503 |
| 1900 | ,, | 150 | ,, | ,, | 330,000 |

In 1892 the central block was enlarged so as to provide administrative accommodation for over 1,000 patients and staff.

It will be seen that with the population of the city at 330,000, the number provided for—namely, 1,000—will be approximately in accordance with the average ratio of one insane in every three hundred people; the exact proportion for England and Wales being in 1901, 1 in 301.32, as compared with 1 in 335 in 1892 and 1 in 536 in 1859.

The new block for 150 women consists, like the rest of the building, of two storeys, but, unlike it, is designed on the separate block system instead of the continuous corridor plan, the advantage being that this contrivance obviates all possibility of two adjoining wards being thrown into one and left in charge of one ward attendant; also it provides better isolation in case of fire or infectious disease.

The architecture is of the same style as that of the older parts of the Institution, being faced with pennant ashlar and finished with Box ground stone dressings. The floors and ceilings are rendered impervious to fire by means of a thick layer of concrete.

Inside, the wards have an artistic appearance: the floors are of American maple, and the furniture is not only comfortable but elegant. As regards air space, there is an allowance of 1,200 cubic feet per patient in the sick ward, and the advantages of a heating apparatus by Hadyn, as well as electric lighting—as in the rest of the building,—add considerably to the comfort of the wards.

The new block includes a general bath-room with six porcelain baths, and at the back, overlooking Purdown, is an annexe for night nurses and sick nurses.

At a distance from the main building is the new infectious hospital, with beds for 12 patients; the building has an administrative block of its own, and is, in fact, self-contained.

There was also opened in 1900 a new dining-hall for male patients. This is a large and lofty room, with good acoustic properties, and with a permanent stage, together with the necessary green-rooms and the usual stage fittings.

